# Predicting cervical lymph node metastasis in OSCC based on computed tomography imaging genomics

**DOI:** 10.1002/cam4.6474

**Published:** 2023-08-27

**Authors:** Nenghao Jin, Bo Qiao, Min Zhao, Liangbo Li, Liang Zhu, Xiaoyi Zang, Bin Gu, Haizhong Zhang

**Affiliations:** ^1^ Medical School of Chinese PLA Beijing China; ^2^ Department of Stomatology, The First Medical Centre Chinese PLA General Hospital Beijing China; ^3^ Pharmaceutical Diagnostics, GE Healthcare Beijing China; ^4^ Research Center of Medical Big Data, Chinese PLA General Hospital Beijing China

**Keywords:** computed tomography imaging (CT imaging)3, genomics4, lymph node metastasis (LNM)2, oral squamous cell carcinoma (OSCC)1, ribonucleic acid sequencing (RNA‐seq)5

## Abstract

**Background:**

To investigate the correlation between computed tomography (CT) radiomic characteristics and key genes for cervical lymph node metastasis (LNM) in oral squamous cell carcinoma (OSCC).

**Methods:**

The region of interest was annotated at the edge of the primary tumor on enhanced CT images from 140 patients with OSCC and obtained radiomic features. Ribonucleic acid (RNA) sequencing was performed on pathological sections from 20 patients. the DESeq software package was used to compare differential gene expression between groups. Weighted gene co‐expression network analysis was used to construct co‐expressed gene modules, and the KEGG and GO databases were used for pathway enrichment analysis of key gene modules. Finally, Pearson correlation coefficients were calculated between key genes of enriched pathways and radiomic features.

**Results:**

Four hundred and eighty radiomic features were extracted from enhanced CT images of 140 patients; seven of these correlated significantly with cervical LNM in OSCC (*p* < 0.01). A total of 3527 differentially expressed RNAs were screened from RNA sequencing data of 20 cases. original_glrlm_RunVariance showed significant positive correlation with most long noncoding RNAs.

**Conclusions:**

OSCC cervical LNM is related to the salivary hair bump signaling pathway and biological process. Original_glrlm_RunVariance correlated with LNM and most differentially expressed long noncoding RNAs.

## 1 INTRODUCTION

Oral cancer accounts for 3%–5% of all systemic malignant tumors. It is the 15th most common malignant tumor in terms of incidence, with more than 480,000 new cases and approximately 220,000 global deaths each year^1^; In this context, oral squamous cell carcinoma (OSCC) accounts for 90% of oral cancers.^2^ After comprehensive treatment, the 5‐year survival rate of OSCC remains at 50%,^3^, ^4^ while that of patients with cervical lymph node metastases (LNM) is only 20%. Ultrasonography (US), computed tomography (CT), magnetic resonance imaging (MRI), and 18F‐FDG PET/CT have been widely used to evaluate the cervical LN status of OSCC patients before surgery.^5^, ^6^, ^7^ Medical imaging plays an important role in the clinical decision‐making process in oncology. Radiomics is used to decode tumor phenotypes by converting conventional imaging images of patients into quantitative data that can be mined, so as to help in the application of tumor diagnosis, efficacy prediction, and prognosis analysis.^8^ Therefore, accurate assess cervical LN status based on CT radiomics, and screening of biomarkers related to cervical LNM are crucial for the treatment and prognosis of OSCC.

Molecular characterization of tumor tissue by genomics and proteomics is achieved by obtaining tissue specimens through invasive surgery. However, specimens have an inherent selection bias during the biopsy process and cannot accurately display the entire lesion.^9^, ^10^ Ribonucleic acid‐sequencing (RNA‐seq) can provide a comprehensive view of the entire, including fusion gene identification, coding sequence polymorphism study, gene expression analysis, directional sequencing, and single‐cell sequencing transcript and shape understanding on genome function from all aspects.^11^

The ideal biomarker is noninvasive, predictive, high spatiotemporal resolution, and inexpensive. With tissue and blood compared with liquid‐derived biomarkers, imaging genomics combines radiographic phenotypes and genome data; it analyzes the relevance of genetic mutations and diagnoses radiographic features in intermediate phenotypes to explore disease pathogenesis. These markers can be used to predict treatment response and the likelihood of early metastasis and to personalize treatment decisions.^12^, ^13^, ^14^ Image genomics has been applied to the study of various types of tumors, including renal clear cell carcinoma,^15^ liver cancer,^16^ and breast cancer^17^ among others. It is noteworthy that standardized image acquisition and reconstruction is an important step to achieve the accuracy and reliability of imaging genomics research and analysis results.^18^ In addition, the Image Biomarkers Standardization Initiative (IBSI) should also be established when calculating and extracting the images required for radiomics features.^19^ Tumor phenotypic characteristics can be obtained through multimodal high‐throughput noninvasively, and radiomics features can be extracted based on standardized imaging data to jointly serve as noninvasive biomarkers for early tumor identification in clinical practice.^20^

This study was aimed to analyze the correlation between imaging and gene expression in patients with cervical LNM from OSCC. Firstly, the tumor‐based multi‐region analysis included multiple quantitative imaging features, the Minimum Redundancy Maximum Relevance(mRMR) and Least absolute shrinkage and selection operator (LASSO) were used to further screen the optimal radiomics features. Weighted Gene Co‐expression Network Analysis (WGCNA) was performed to capture the gene expression patterns of lymph node metastasis in patients with OSCC. It also screened the association between LNM radiomic features and gene modules, explored imaging biomarkers and pathways of LNM, and aimed to predict the development of LNM in patients with OSCC.

## 2 MATERIALS AND METHODS

### 2.1 Screening of CT radiomic features of OSCC

**FIGURE 1:**
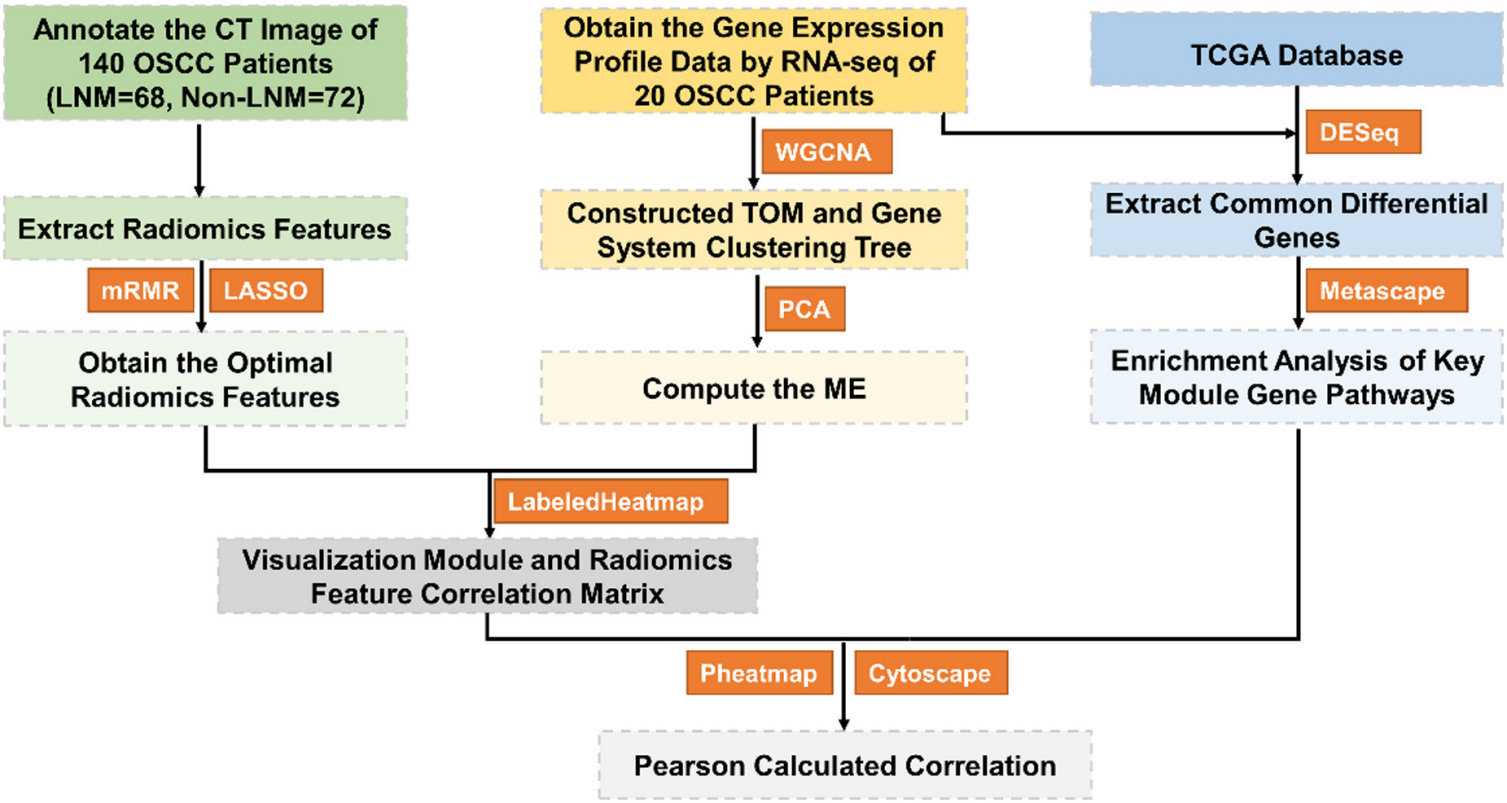
Work flowchart for the study. TCGA, the cancer genome atlas; OSCC, oral squamous cell carcinoma; non‐LNM, non lymph node metastasis; LNM, lymph node metastasis; LASSO, least absolute shrinkage and selection operator; WGCNA, weighted gene co‐expression network analysis; mRMR, minimum redundancy maximum relevance.

The study was approved by the Ethics Committee of the Chinese PLA General Hospital. A total of 140 patients with OSCC who underwent enhanced CT scan in the Department of Oral and Maxillofacial Surgery, Chinese PLA General Hospital from January 2014 to June 2020 were retrospectively analyzed for CT radiomics feature extraction (Table S1). Twenty randomly selected tumor tissue samples were used for RNA‐seq (Table S2). Patients who fulfilled the following criteria were included: no history of preoperative radiotherapy, chemotherapy, or any other anticancer therapy; underwent definitive surgery with a clear postoperative pathological diagnosis of squamous cell carcinoma. ITK‐SNAP software (version 3.8.0, University of Pennsylvania, www.itksnap.org) was used to determine and delineate the region of interest (ROI) at the edge of the primary tumor on CT images. The PyRadiomics library from python was used to extract radiomic features from the delineated ROI. Finally, mRMR and LASSO were used to further select the optimal radiomic features (Figure 1).

### 2.2 RNA‐seq

For sample testing, 10–1000 ng/μL of total RNA with an RNA integrity number of ≥7.0 and 28S/18S ribosomal RNA (rRNA) ≥1.0 was analyzed using an Agilent 2100 Bioanalyzer. Following digestion with deoxyribonuclease I, a Ribo‐Zero TM kit was used for removing the rRNA from the total RNA, and the messenger RNA (mRNA), long noncoding RNA (lncRNA), and circular RNA were purified. The mRNA was broken into 140–160 nt fragments using a fragmentation buffer, and the secondary structure was denatured and hybridized with primers. A strand of circular deoxyribonucleic acid was synthesized using six‐base random primers by polymerase chain reaction (PCR). Double‐stranded circular deoxyribonucleic acid was synthesized from deoxyuridine triphosphate, and the ends were repaired using a Thermomixer Comfort apparatus by adding adenine bases to the 3′‐end. Following PCR amplification, an Agilent 2100 Bioanalyzer was used for detecting the range of the inserted fragments in the library. An ABI StepOnePlus Real‐Time PCR System (TaqMan Probe) was used to measure the quantitative concentration of the library. The library was denatured into single strands by the addition of sodium hydroxide, and hybridized with the connector on a Flow Cell. Bridge PCR amplification was performed on a cBot system; sequencing was finally performed on a DNBSEQ platform. The low‐quality reads containing adapter sequences and reads with >10% N (number of bases with mass values ≤5 accounting for >50% of the total reads) were removed. The gene expression profile data were then obtained by quality control of the raw sequencing data using FastQC software.

### 2.3 Differential expression analysis

DESeq software was used for analyzing differential gene expression patterns in the expression profile data; the screening threshold was as follows: *p* < 0.05 and |log2FoldChange| > 1. The VennDiagram package (version 1.6.20) in R (version 4.0.1) was used to depict the differentially expressed genes in the 20 OSCC samples and the Cancer Genome Atlas (TCGA) database. The differentially expressed genes that were common between the two groups were subsequently extracted.

### 2.4 Weighted gene co‐expression network analysis (WGCNA)

The differential gene expression data were normalized to transcript per million values to ensure comparability of the gene expression profiles among the samples. The optimal act law coefficient β value was determined based on the pickSoftThreshold function in the WGCNA package (version 1.69).

The gene expression similarity matrix was converted into a network, constructed based on the adjacency matrix. The degree of dissimilarity between the genes was subsequently calculated and converted into a topological matrix. The topological overlap matrix was used to describe the level of association between the genes, and the hierarchical clustering method was used to obtain a gene clustering tree. The modules were split with the dynamic clipping tree algorithm and the minimum number of genes in each module was set to 30. The modules with similar expression were merged using the parameters TOMType = “unsigned” and MergeCutHeight = 0.25, and the network was finally constructed.

The module eigengene (ME) was calculated by principal component analysis of the gene expression matrix of the entire module. The Pearson correlation coefficient between the ME and radiomic features was then calculated for determining the relationship between the modules and radiomic features. Smaller *p* values were indicative of more significant correlation. The labeledHeatmap function was used to visualize the model and the above correlation matrix.

### 2.5 Enrichment analysis of key module gene pathways

Metascape (http://metascape.org/) was used for online analysis of the shared differentially expressed genes identified from the sequencing data and TCGA database for the 20 OSCC samples in this study. Bar charts depicting the results of gene functional pathway enrichment were also prepared using Metascape. The genes that demonstrated significant correlation with the radiomic features were extracted. The genes were annotated by Gene Ontology (GO) analysis, into biological process (GO_BP), molecular function (GO_MF), and cellular component (GO_CC) categories. The Kyoto Encyclopedia of Genes and Genomes (KEGG) database was used for classification and annotation of the genes. The results of KEGG functional enrichment were depicted using bubble charts.

### 2.6 Correlation analysis

Pearson correlation analysis was used for determining the correlation between the major enriched pathway genes and radiomic features in the key gene modules; these were visualized using the Pheatmap package (version 1.0.12) and Cytoscape (version 3.7.2), respectively.

## 3 RESULTS

### 3.1 Radiomic feature screening

**FIGURE 2:**
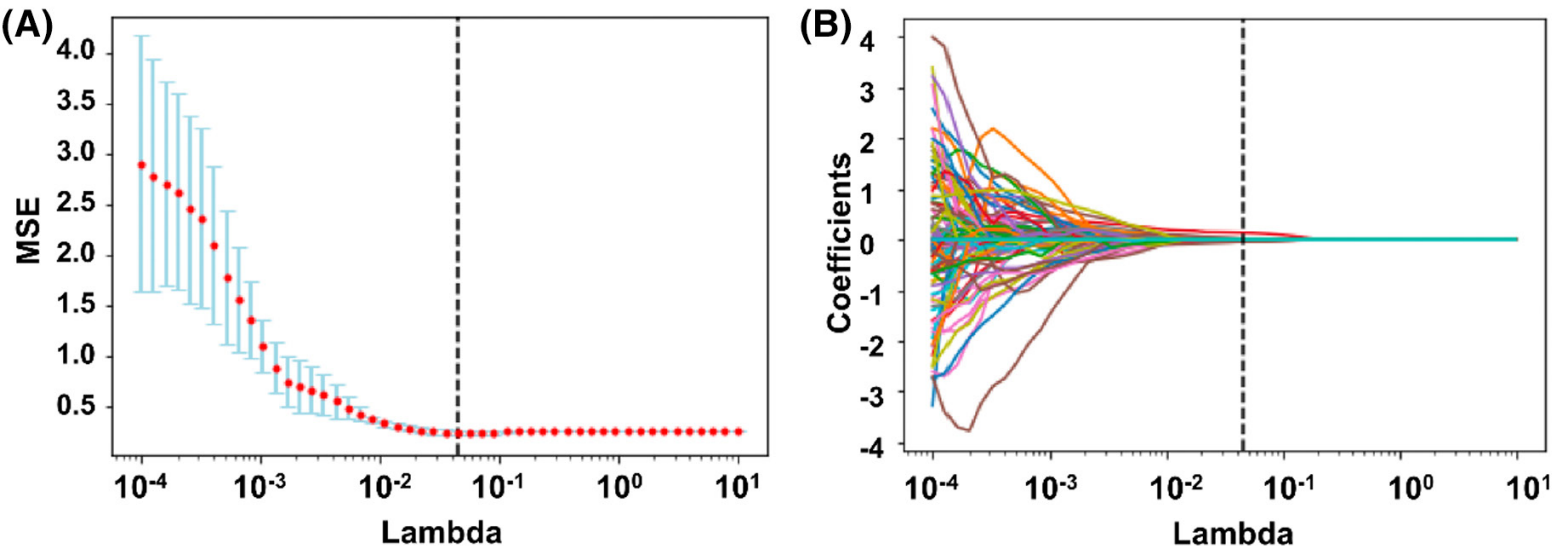
Screening features in LASSO regression. (A) Relationship between the adjustment parameter (lambda) and mean square error (MSE) in LASSO regression; (B) Trend plot of the adjustment parameter (lambda) for 171 radiomic features in LASSO regression.

A total of 480 radiomic features were extracted from 140 patients with OSCC. The mRMR algorithm was used to further screen 171 features for LASSO analysis (Figure 2). LASSO regression was used to select the optimized feature subset. The seven following radiomic features were selected: Original_shape_Maximum2DDiameterRow, original_glrlm_RunVariance, original_glszm_Zonepercentage, square_firstorder_Median, square_glrlm_ShortRunHighGrayLevelEmphasis, logarithm_glszm_SmallAreaEmphasis, and exponential_firstorder_RootMeanSquared.

### 3.2 Differential gene analysis of pathological samples from patients with OSCC

**FIGURE 3:**
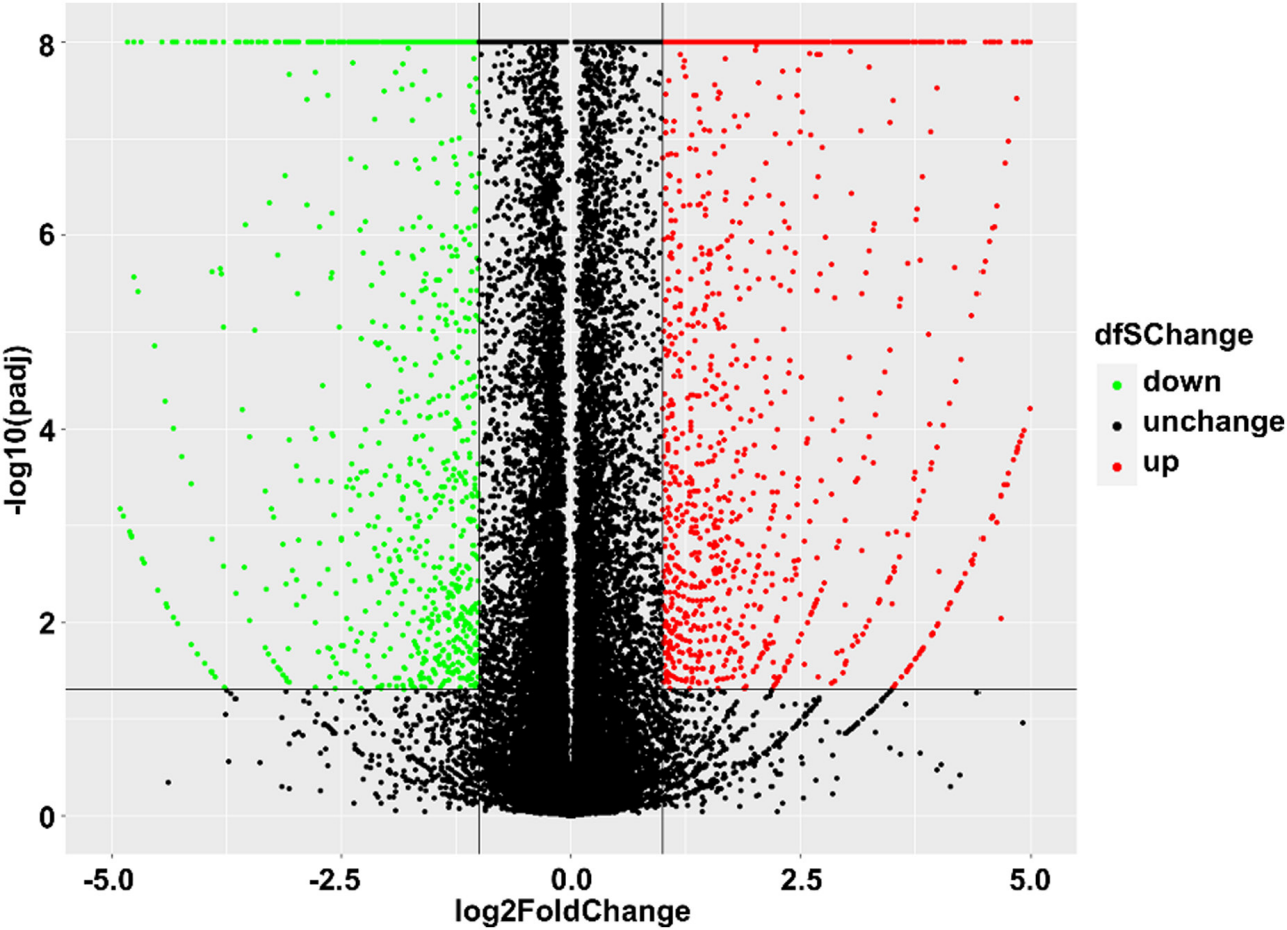
Volcano plot depicting the overall distribution of the differentially expressed genes. For the DERNAs between the LNM and non‐LNM groups, the 2157 upregulated genes are depicted in red and the 1370 downregulated genes are depicted in green. Genes with no significant difference in expression levels are depicted in black.

The transcriptome sequencing RNA‐seq data of the 20 patients in the experimental group revealed a total of 3527 differentially expressed RNAs (DERNAs) between LNM and non‐LNM; 2157 genes were upregulated while 1370 were downregulated (Figure 3). In the control setup, a total of 139 differentially expressed genes were screened between the LNM and non‐LNM groups in TCGA database. Among them, 41 genes were differentially expressed in both datasets (Figure S1). Gene were primarily enriched in the GO: 0031424 (keratinization) pathway term (Figure S2). The common differential genes that were enriched in this pathway included GAL, KRT81, TGM3, LCE2A, FOXJ1, SPRR3, LCE2B, and LCE2D (Table 1).

**TABLE 1:** Enrichment of common differential genes in keratinization.

Gene symbol	Description	Biological process (GO)	Protein function (Protein Atlas)
GAL	Galanin and GMAP prepropeptide	GO:0051795 positive regulation of timing of catagen	Disease related genes; Predicted secreted proteins
		GO:1902606 regulation of large conductance calcium‐activated potassium channel activity	
		GO:1902608 positive regulation of large conductance calcium‐activated potassium channel activity	
KRT81	Keratin 81	GO:0070268 cornification GO:0031424 keratinization GO:0030216 keratinocyte differentiation	Disease related genes Predicted intracellular proteins
TGM3	Transglutaminase 3	GO:0043163 cell envelope organization	Disease related genes ENZYME proteins; Transferases
		GO:0045229 external encapsulating structure organization	Enzymes Potential drug targets;
		GO:0031069 hair follicle morphogenesis	Predicted intracellular proteins
LCE2A	Late cornified envelope 2A	GO:0031424 keratinization; GO:0030216 keratinocyte differentiation GO:0009913 epidermal cell differentiation	Predicted intracellular proteins
FOXJ1	Forkhead box J1	GO:0002510 central B cell tolerance induction GO:0002646 regulation of central tolerance induction GO:0002648 positive regulation of central tolerance induction	Predicted intracellular proteins Disease related genes Cancer‐related genes Candidate cancer biomarkers; Transcription factors; Helix‐turn‐helix domains
SPRR3	Small proline rich protein 3	GO:0018149 peptide cross‐linking	Predicted intracellular proteins
		GO:0070268 cornification	Cancer‐related genes Candidate cancer biomarkers
		GO:0031424 keratinization	
LCE2B	Late cornified envelope 2B	GO:0031424 keratinization	Predicted intracellular proteins
		GO:0030216 keratinocyte differentiation	
		GO:0009913 epidermal cell differentiation	
LCE2D	Late cornified envelope 2D	GO:0031424 keratinization;	Predicted intracellular proteins
		GO:0030216 keratinocyte differentiation	
		GO:0009913 epidermal cell differentiation	

### 3.3 Construction of gene co‐expression network module

WGCNA is used to simplify complex gene expression data into several functional modules by measuring the co‐expression relationship between gene expression correlation coefficients and radiomic features; the modules with biological significance can be identified by combining the phenotypic information. The optimal act law coefficient β value was determined to be 26; this indicates that the network was scale‐free (Figure S3).

**FIGURE 4:**
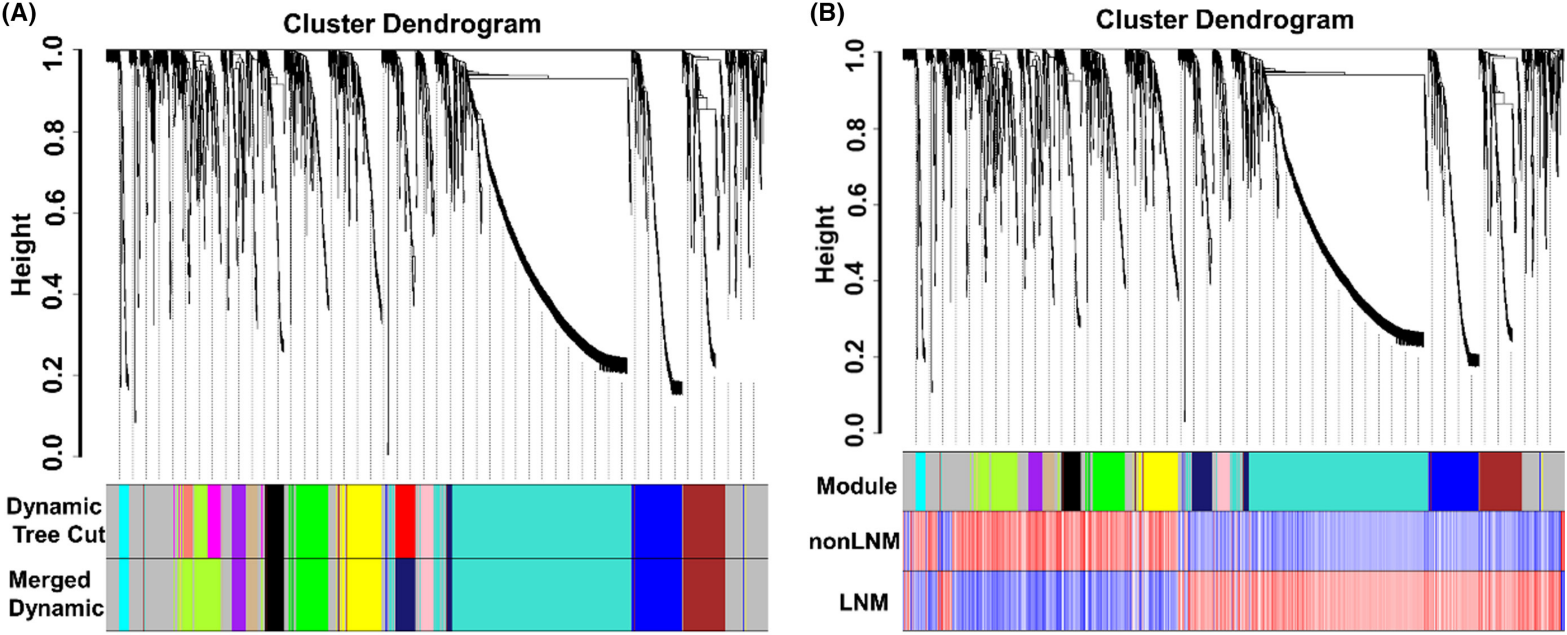
Construction of the co‐expressed gene network module and verification of gene expression levels. (A) The upper part of the co‐expressed gene network module is divided into clustering trees, the middle color bar is the gene module segmented by the dynamic shear tree algorithm, and the lower color band is the final gene module result after merging the modules with similar expression. (B) Relative expression levels of LNM and non‐LNM genes; red represents high expression and blue represents low expression.

The modules with similar expression were merged and a total of 13 gene modules were finally constructed (Figure 4A). The largest module comprised 1013 RNAs (gene module in turquoise), while the smallest module contained 49 RNAs (gene module in cyan). The number of genes in each module has been depicted in the Table S3. The relative expression levels of the genes in the LNM and non‐LNM groups were subsequently determined (Figure 4B). In the largest gene module, the genes in the LNM group were relatively highly expressed, while the genes in the non‐LNM group exhibited relatively low expression.

**FIGURE 5:**
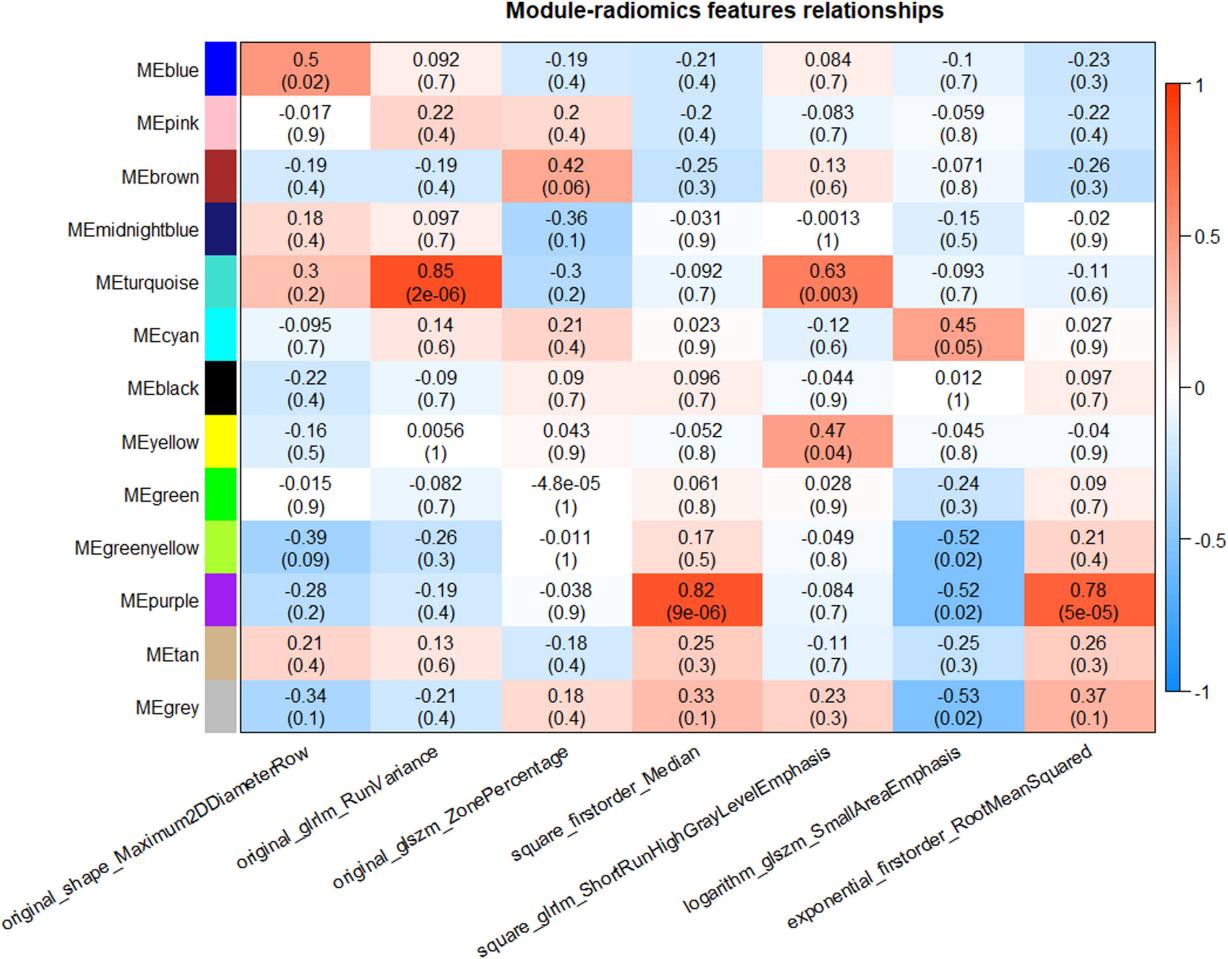
Heat map of correlation between gene modules and radiomic features. Rows represent the module eigengene (ME) of each module and represent the overall expression level of the module. Columns represent radiomic features. Each square is represented by the r value (*p* value); r is the correlation coefficient between modules and radiomic features and the *p* value represents the significance of the correlation coefficient.

The heat map of correlation between gene modules and radiomic features (Figure 5) showed that the original_glrlm_RunVariance feature had the strongest correlation with the turquoise gene modules (correlation coefficient 0.85, *p* < 0.001). The correlation between the purple gene module and the square_firstorder_Median feature was second only to that with square_firstorder_Median (correlation coefficient 0.82, *p* < 0.001). The exponential_firstorder_RootMeanSquared feature also showed correlation with the purple gene module (correlation coefficient 0.78, *p* < 0.001). The turquoise and purple gene modules were screened for subsequent analysis.

### 3.4 Enrichment analysis of key gene module pathways

**FIGURE 6:**
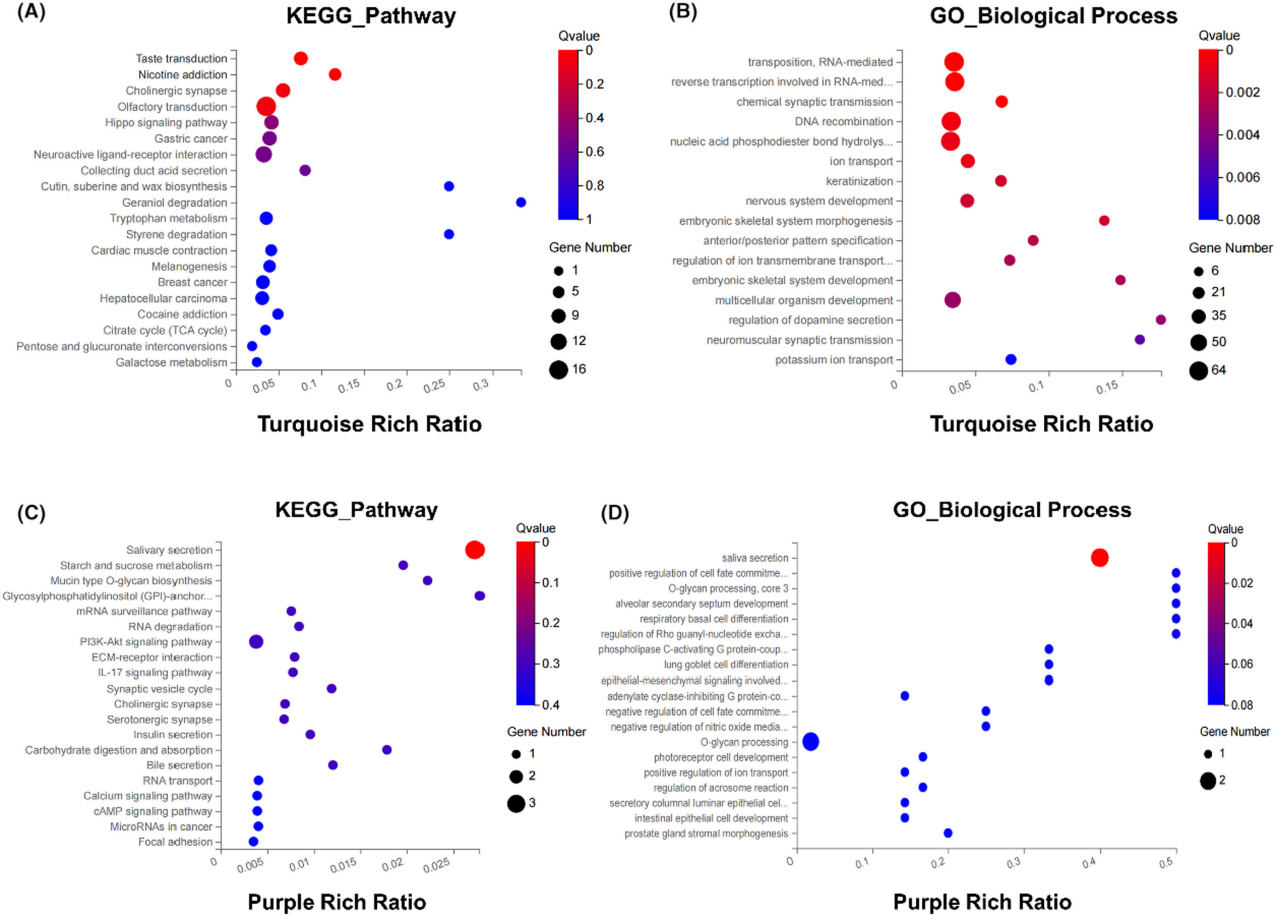
Enrichment bubble diagram. Enrichment bubble diagram of Turquoise gene module based on KEGG_Pathway (A), GO_BP (B); Purple module gene based on KEGG_Pathway (C); GO_BP (D).

The turquoise gene modules were mainly enriched in the “taste transduction” signaling pathway of the KEGG database (Figure 6A) and the “transposition, RNA‐mediated” biological process of GO_BP (Figure 6B). In the KEGG database(Figure 6C), the purple gene module was mainly enriched in the salivary secretion pathway; in GO_BP, it was mainly enriched in the biological process of saliva secretion (Figure 6D). Moreover, the enrichment bubble plots of the two genes in GO_CC and GO_MF are shown in Figure S4, respectively.

### 3.5 Correlation analysis between genes and radiomic features

**FIGURE 7:**
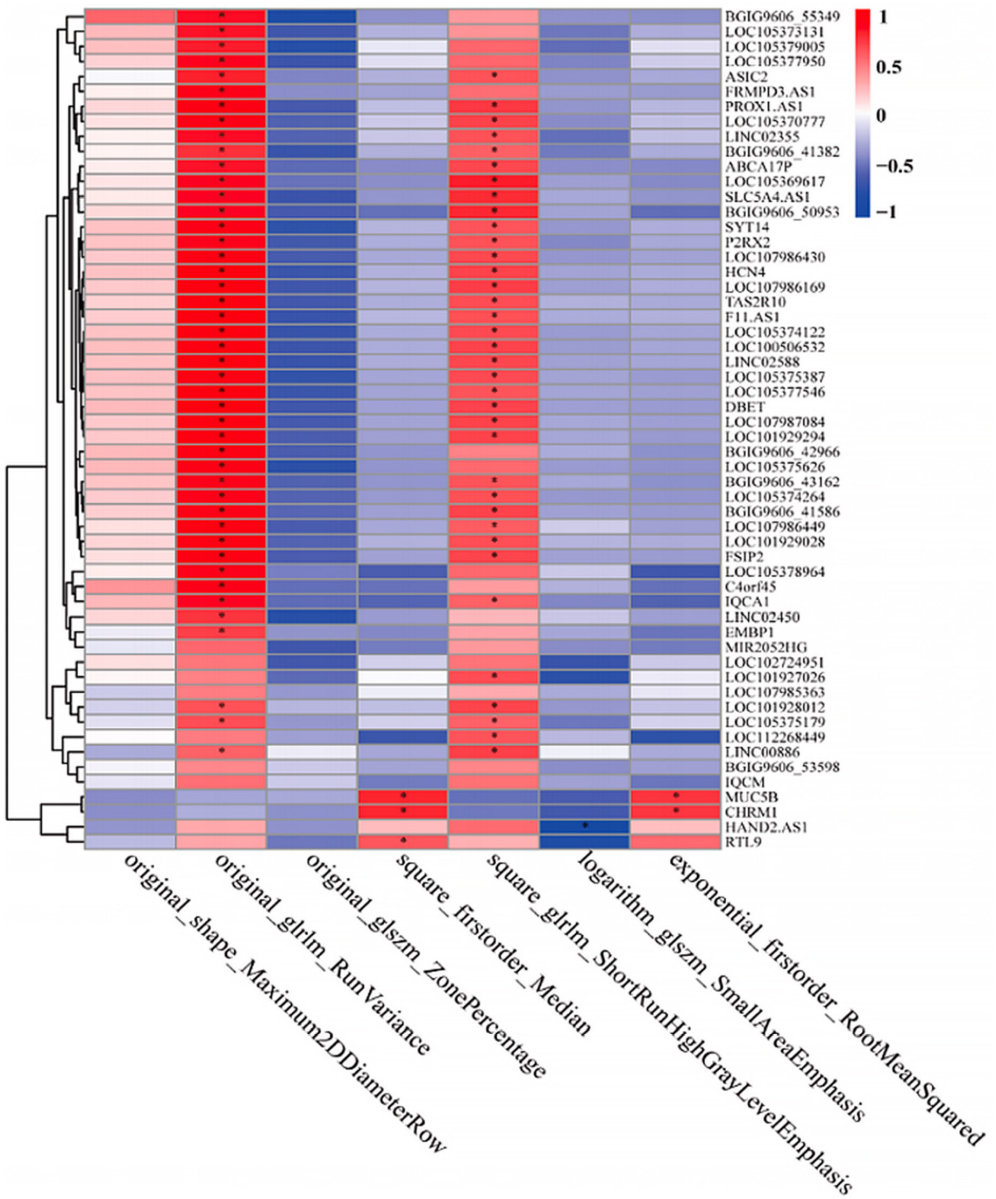
Correlation between key genes and the radiomic features heat map. Rows represent key genes, columns represent radiomic features, red represents positive correlation, and blue represents negative correlation, * represents significance (*p* < 0.05).

**FIGURE 8:**
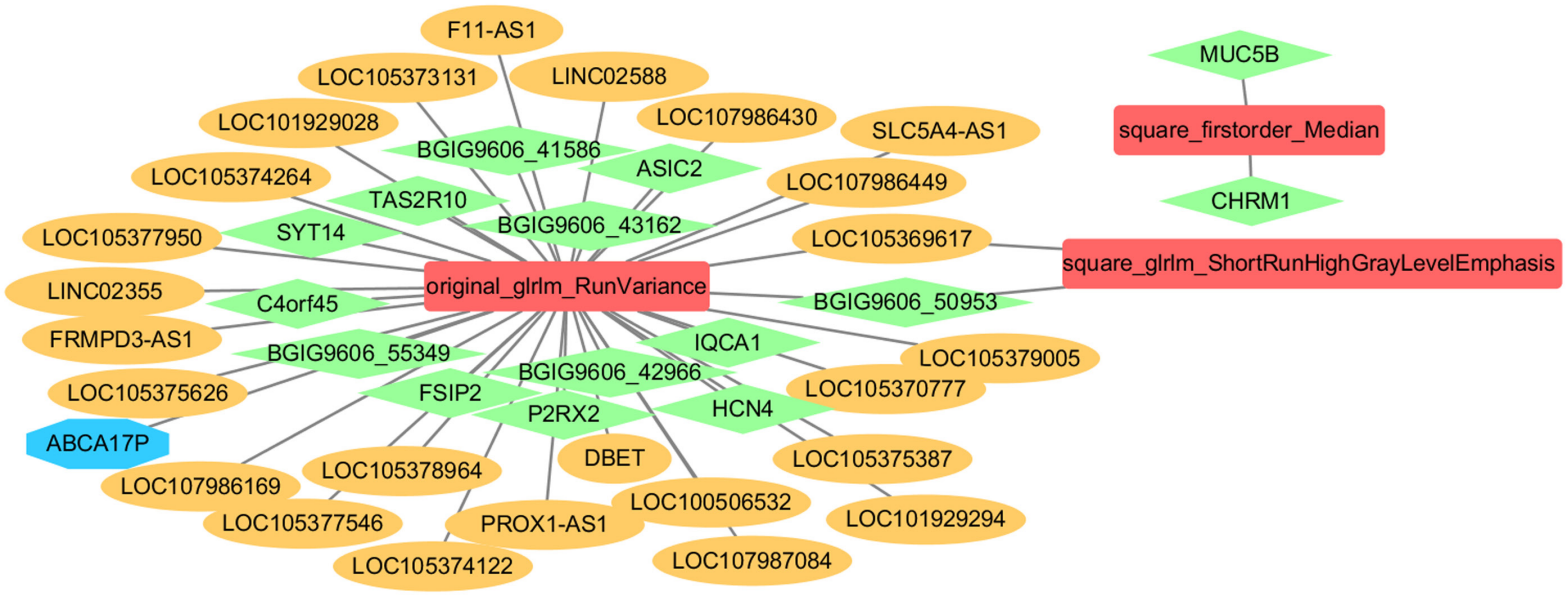
Correlation network diagram between key genes and radiomic features (lncRNAs in orange, mRNAs in green, pseudo genes in blue, and radiomic features in red). The lines represent correlations and solid lines represent positive correlations.

A total of 56 genes were extracted from the main enrichment pathways of the turquoise and purple gene modules. The correlation between each gene and radiomic eigenvalue was calculated based on the expression levels of each gene. The results showed significant positive correlation (*p* < 0.05) between most genes and the two image features, square_glrlm_ShortRunHighGrayLevelEmphasis and original_glrlm_RunVariance (Figure 7). The correlation network diagram between key genes and radiomic features, which was constructed based on screening, showed that the original_glrlm_RunVariance feature correlated significantly and positively with most long noncoding RNAs (Figure 8).

## 4 DISCUSSION

The concept of imaging genomics was first proposed by the European Society for Radiotherapy and Oncology (ESTRO) to establish correlations between gene expression data and radiomic features.^21^ In a study from Israel, Segal et al^16^ obtained 28 image features and gene expression profiles of patients with primary liver cancer after contrast enhanced CT scanning; they proposed a three‐step strategy for performing correlation analysis between radiomic features and gene expression. Image genomics correlates quantitative or qualitative imaging features with genomic data obtained from tissue analysis and other clinical data to provide more comprehensive understanding on tumors; this in turn aids the identification of imaging biomarkers that can be used as alternatives to genetic testing. The noninvasive nature and high spatial and temporal resolution of imaging biomarkers make them ideal for therapeutic monitoring and predictive analysis.

Although studies performed over the past few decades have elucidated some of the mechanisms underlying OSCC progression, most studies have focused on protein‐coding genes, with few studies based on noncoding RNA. Protein‐coding genes comprise only 2% of the human genome, and most transcripts are noncoding RNA, including lncRNAs, which exceed 200 nt in length and do not have protein‐coding ability. Studies have shown that genomic regions that cannot encode proteins play an important role in regulating cellular physiological processes and disease progression; these regions are usually transcribed as lncRNA.^22^, ^23^, ^24^ Based on the correlation between gene and radiomics features greater than 0.75. In this study, the radiomic feature, ‘original_glrlm_RunVariance’, which was extracted from enhanced CT images of 140 patients with OSCC, correlated significantly and positively with various lncRNA including F11‐AS1, LOC105373131, and LINC02588. A large number of abnormally expressed lncRNAs are associated with various cancers, and have become an important universal gene category in cancer development and progression. OSCC demonstrates many instances of gene regulation by lncRNAs; these are associated with and affect various aspects of cell balance including proliferation, survival, migration, and genomic stability. There is currently no OSCC‐specific diagnostic marker. Abnormal expression of some lncRNAs has been shown to be closely related to cancer prognosis. HOX transcript antisense RNA (HOTAIR) is highly expressed in OSCC tissues, and its expression level correlates with tumor size, TNM stage, and prognosis. These findings suggest that HOTAIR can be used as a biomarker for the diagnosis and prognostication of OSCC and as a molecular target for treatment.^25^ Tang et al^26^ found that MALAT‐1 and HOTAIR are expressed in patients with primary OSCC tumors; the expression level of HOTAIR differed between patients with LNM and primary tumor controls. This suggests that lncRNA detection offers promise for the noninvasive and rapid diagnosis of OSCC in the clinic; it may also help determine the presence of cervical LNM. The therapeutic effect and possibility of early cervical LNM can therefore be further predicted based on these imaging features.

Many genomic and clinical biomarkers identified from various types of cancer have been included in TCGA; these correlate with corresponding images in the Cancer Imaging Archive database.^14^ However, imaging data from the Cancer Imaging Archive (TCIA) cannot be used in clinical practice as the results of genetic testing cannot be matched with specific locations on images.^10^ TCIA database was different from the imaging data types of 140 patients with OSCC in this study. contrast‐enhanced CT was used in 140 patients with OSCC, while Unenhanced CT was used in TCIA database. Imaging genomics can quantify lesion characteristics, better distinguish tumor nature and metastasis risk, stratify patients according to risk, and allow more accurate imaging and screening of tumors.^27^, ^28^ Zhou et al^29^ analyzed preoperative CT imaging features in 113 cases of non‐small cell lung cancer and detected changes in genetic information from tumor tissues by RNA‐seq; they then identified the genes associated with ground‐glass shaped irregular nodules. Using radiomics and gene expression analysis of enhanced CT images, Badic et al^30^ found that enhanced CT combined with gene expression can predict the prognosis of primary colorectal cancer more effectively; this is conducive to the formulation of treatment plans.

The main limitations of current imaging genomics models include the lack of reproducibility and stability.^31^ In this study, owing to the small sample size (as in most retrospective studies), a prospective validation cohort is lacking. The main limitation of deep learning‐based image genomics is the availability of a limited data set. Insufficient volumes of required data lead to insufficient stratification between training, validation, and test queues.^32^, ^33^, ^34^ Interpretable models therefore need to be urgently combined with open access and high‐quality public databases, as well as complete genomic and imaging data for different types of tumors; this will allow tumor heterogeneity to be better studied and addressed. We contrast enhanced CT images of cervical LNM of OSCC were delineated according to this standard, and the imaging features were obtained, and finally screened a small number of reliable mark genes related to radiomic features. And finally screened a small number of reliable mark genes related to radiomic features. In addition, factors such as different cancer cell clones, tumor microenvironment, cell cycle and cell differentiation should be taken into account when establishing radiogenomics models,^35^ although these factors may eventually be replaced by more effective radiogenomics.

In summary, with the development of precision medicine, image genomics has become a bridge between the tumor phenotype and genotype. From the perspective of cancer management, the role of imaging genomics is expected to expand across all stages, including tumor diagnosis, treatment response prediction, and risk monitoring. Imaging genomics not only provides more accurate prediction results, but also more effective treatment options. The use of imaging biomarkers to reflect gene expression analysis of solid tumors provides a rapid and reproducible noninvasive evaluation modality for more accurate diagnosis and prediction through detailed analysis of images. Further studies are needed to explore the association between radiomic features and key genes; this will allow researchers to determine whether new biomarkers can predict diagnosis, metastasis, or treatment response in independent patient populations. It will also improve the accuracy of prognostication and help develop the best treatment plan, thereby contributing to personalized precision therapy.

## 5 CONCLUSIONS

CT imaging of cervical lymph node metastasis in OSCC is closely related to RNA sequencing. Based on RNA‐seq data analysis from OSCC patients, the findings from this study suggest that the “salivary secretion” signaling pathway and biological process may be related to the development of LNM in OSCC. Correlation analysis of radiomic features and key genes extracted from enhanced CT showed the “original_glrlm_RunVariance” feature to be related to metastasis; it also showed significant positive correlation with most differentially expressed lncRNAs. This forms a basis for the future search of gene imaging substitutes and the interpretation of radiomic features at the gene level.

## AUTHOR CONTRIBUTIONS

**Nenghao Jin:** Data curation (lead); writing – original draft (lead). **Bo Qiao:** Data curation (equal); investigation (equal). **Min Zhao:** Methodology (equal); software (lead). **Liangbo Li:** Data curation (equal). **Liang Zhu:** Data curation (equal). **Xiaoyi Zang:** Data curation (equal). **Bin Gu:** Funding acquisition (lead); writing – review and editing (equal). **Haizhong Zhang:** Software (equal); writing – review and editing (equal).

## FUNDING INFORMATION

This work were supported by Beijing Natural Science Foundation(Number: Z200001) and the National Key R&D Program of China (No. 2020YFC2008900).

## CONFLICT OF INTEREST STATEMENT

The authors declare that the research was conducted in the absence of any commercial or financial relationships that could be construed as a potential conflict of interest.

## ETHICS STATEMENT

The studies involving human participants were reviewed and approved by Ethics Office of Chinese PLA General Hospital(Number: S2022‐203‐01). And the consent to participate in the study have been obtained from participants. Author confirm that all the experiments on the use of human tissue samples confirm that all experiment were performed in accordance with the Declaration of Helsinki's guidelines and regulations. The study was approved and the participants gave their informed consent.

## CONSENT

Not applicable.

## Supporting information


**Supplementary Figure 1** Venndiagram of the common differential genes in RNA‐seq and TCGA databases of 20 OSCC patients, blue circles are the differential genes between LNM and non‐LNM groups of OSCC patients in TCGA database, yellow circles are the differential genes obtained by RNA‐seq analysis of pathological samples of 20 OSCC patients in experimental group. A total of 41 genes were differentially expressed in both datasets.
**Supplementary Figure 2** Enrichment analysis of common differential gene pathways showed that genes were mainly enriched in the GO: 0031424 (Keratinization) pathway
**Supplementary Figure 3** Selection of parameters for network construction. A. The β values are presented on the x‐axis and the R^2^ values (using the scale‐free topology junction model under different β values) are presented on the y‐axis; B. The x‐axis represents the β values and the y‐axis represents the average adjacency coefficient under different β values.
**Supplementary Figure 4** Enrichment bubble diagram: Enrichment bubble diagram of Turquoise gene module based on GO_CC (A) and GO_MF (B); Purple module gene based on GO_CC (C) and GO_MF (D).
**Supplementary Table 1** Information of 140 patients with oral squamous cell carcinoma
**Supplementary Table 2** The correlation of OSCC clinical‐pathological variables
**Supplementary Table 3** 13 color genes Number of genes contained in the color module

## Data Availability

Relevant data can be obtained by contacting the corresponding author.
