# Supplementary material for: Predicting cervical lymph node metastasis in OSCC based on computed tomography imaging genomics
Source: Cancer Med. 2023 Aug 27;12(18):19260–71. doi: 10.1002/cam4.6474 (PMC10557859; doi:10.1002/cam4.6474)

Supplementary Material

## Supplementary Figures


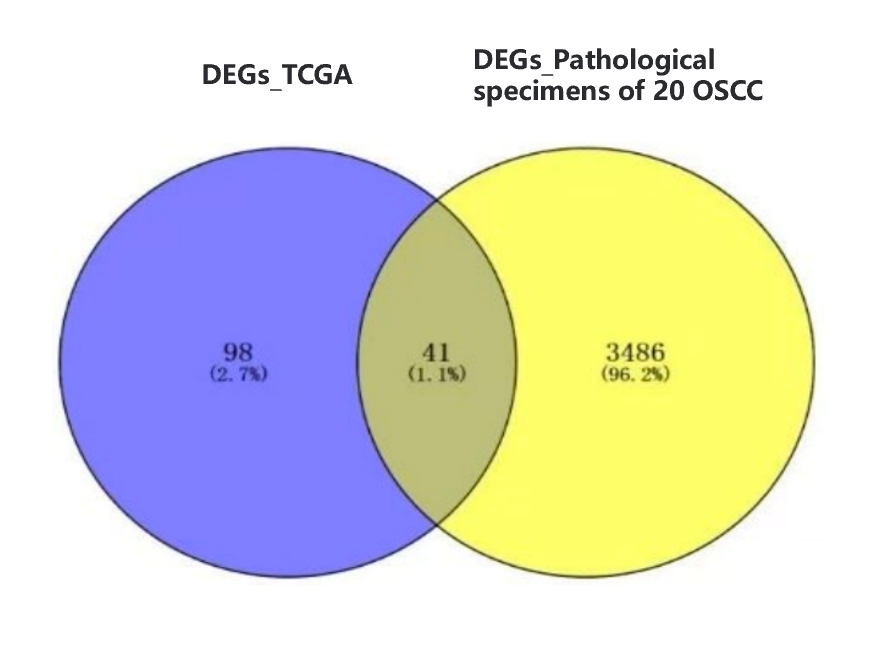


**Supplementary Fig.1** Venndiagram of the common differential genes in RNA-seq and TCGA databases of 20 OSCC patients, blue circles are the differential genes between LNM and non-LNM groups of OSCC patients in TCGA database, yellow circles are the differential genes obtained by RNA-seq analysis of pathological samples of 20 OSCC patients in experimental group. A total of 41 genes were differentially expressed in both datasets.


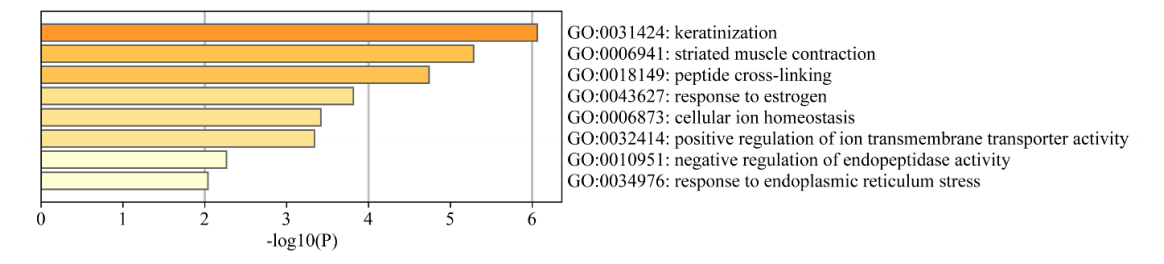


**Supplementary Fig.2** Enrichment analysis of common differential gene pathways showed that genes were mainly enriched in the GO: 0031424 (Keratinization) pathway


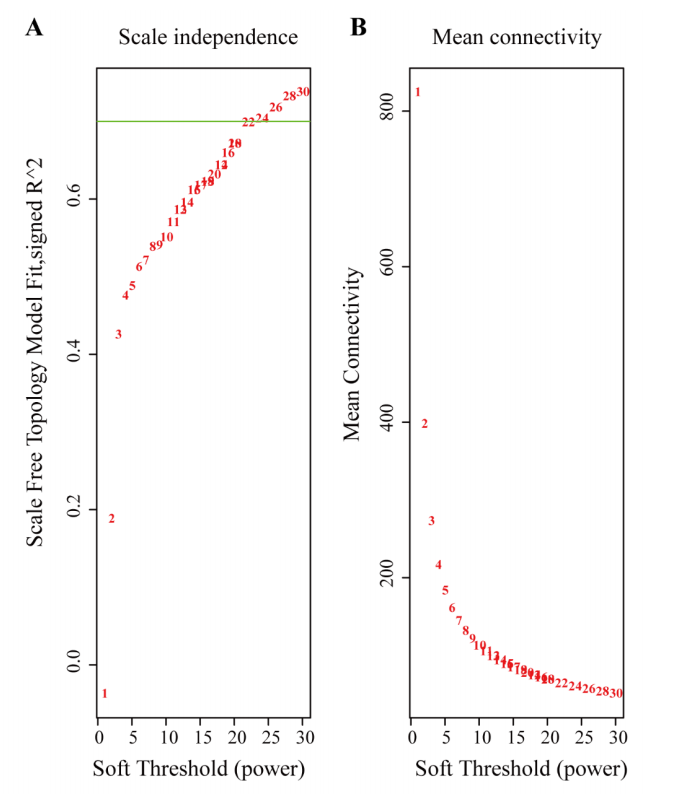


**Supplementary Fig.3** Selection of parameters for network construction. A. The β values are presented on the x-axis and the R^2^ values (using the scale-free topology junction model under different β values) are presented on the y-axis; B. The x-axis represents the β values and the y-axis represents the average adjacency coefficient under different β values.

**
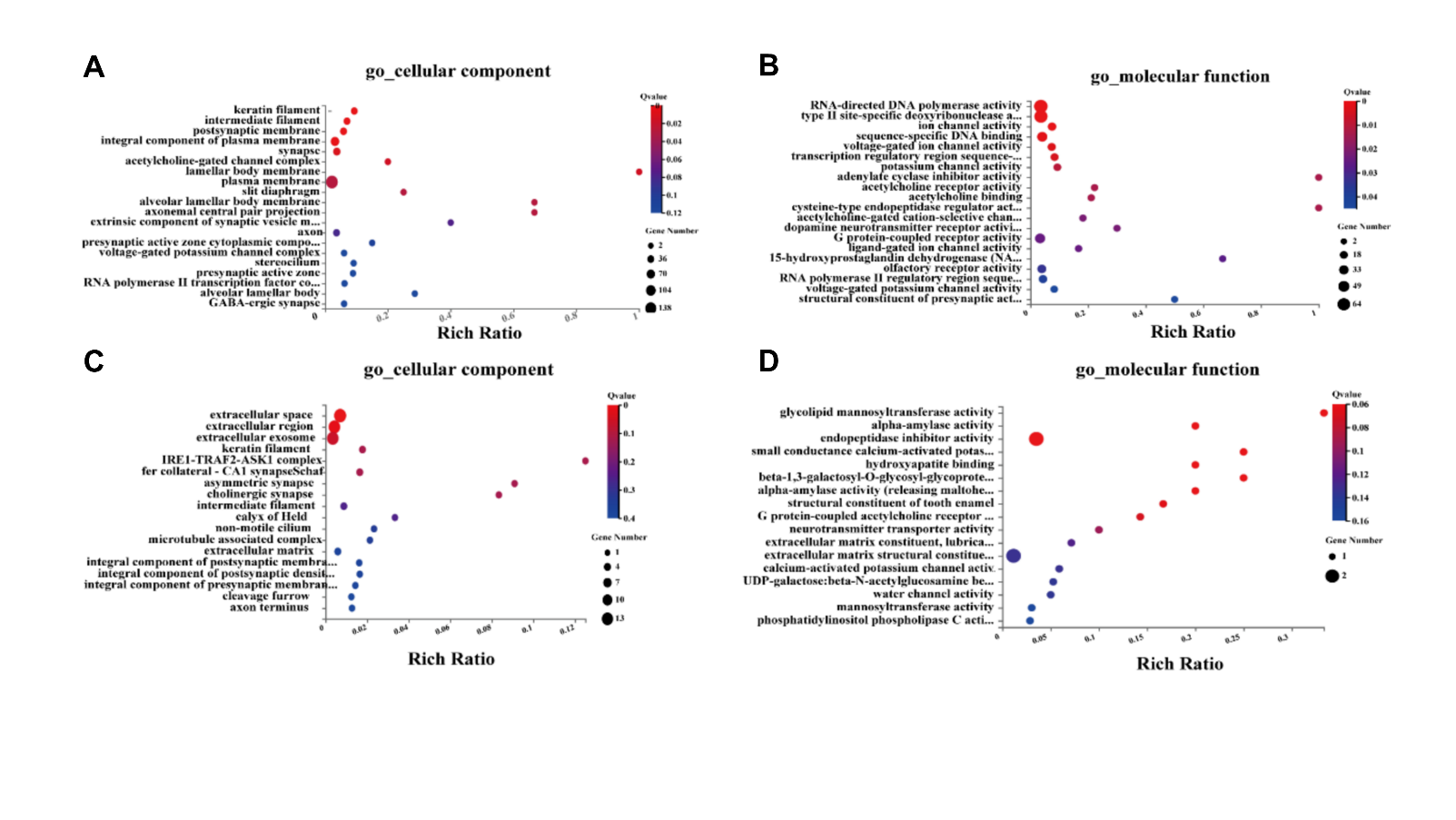
**

**Supplementary Fig.4** Enrichment bubble diagram: Enrichment bubble diagram of Turquoise gene module based on GO_CC (A) and GO_MF (B); Purple module gene based on GO_CC (C) and GO_MF (D).

## Supplementary Tables

**Supplementary Table 1** Information of 140 patients with oral squamous cell carcinoma

| Variable | OSCC with LNM  Patients (n=68) | OSCC with Non-LNM Patients (n=72) |
| --- | --- | --- |
| Age (years, median) | 65.11±12.93 | 57.57±11.31 |
| Gender (n) male/female | 43/25 | 52/20 |
| Location |  |  |
| Tongue | 31 | 37 |
| Gingival | 11 | 13 |
| Buccal mucosa | 7 | 5 |
| Bottom of the mouth | 14 | 13 |
| Palate | 5 | 4 |
| Tumor size stage from CT |  |  |
| T1+T2 | 46 | 58 |
| T3+T4 | 22 | 14 |
| Lymph node status from CT |  |  |
| N0 | 20 | 48 |
| N1+N2 | 48 | 24 |

**Supplementary Table 2** The correlation of OSCC clinical-pathological variables

| Variable | Patients (n=20) |
| --- | --- |
| Age (years, median) | 56.65±14.45 |
| Gender (n) male/female | 15/5 |
| Location |  |
| Tongue | 10 |
| Gingival | 5 |
| Buccal mucosa | 3 |
| Bottom of the mouth | 2 |
| Clinical stage  Ⅰ+Ⅱ | 5 |
| Ⅲ+Ⅳ | 15 |
| Tumor size stage |  |
| T1+T2 | 6 |
| T3+T4 | 14 |
| Nodal status stage |  |
| N0 | 9 |
| N1+N2 | 11 |
| Metastasis stage |  |
| M0 | 20 |
| Histopathological grade |  |
| High grade | 10 |
| Moderate grade | 5 |
| Low grade | 5 |

**Supplementary Table 3** 13 color genes Number of genes contained in the color module


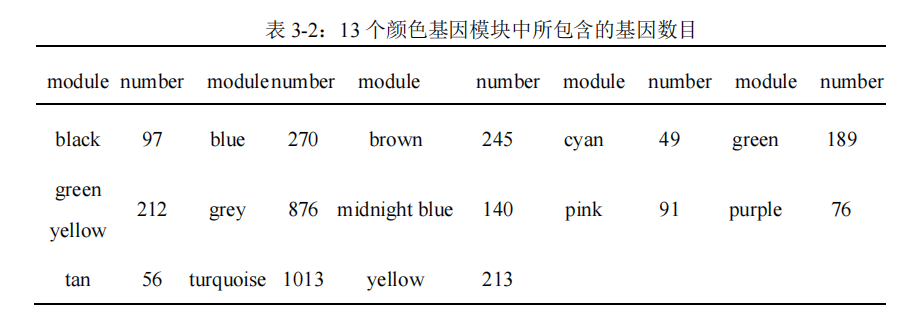

Supplement: Supplementary file 1 — Supplementary Figure 1 Venndiagram of the common differential genes in RNA‐seq and TCGA databases of 20 OSCC patients, blue circles are the differential genes between LNM and non‐LNM groups of OSCC patients in TCGA database, yellow circles are the differential genes obtained by RNA‐seq analysis of pathological samples of 20 OSCC patients in experimental group. A total of 41 genes were differentially expressed in both datasets. Supplementary Figure 2 Enrichment analysis of common differential gene pathways showed that genes were mainly enriched in the GO: 0031424 (Keratinization) pathway Supplementary Figure 3 Selection of parameters for network construction. A. The β values are presented on the x‐axis and the R2 values (using the scale‐free topology junction model under different β values) are presented on the y‐axis; B. The x‐axis represents the β values and the y‐axis represents the average adjacency coefficient under different β values. Supplementary Figure 4 Enrichment bubble diagram: Enrichment bubble diagram of Turquoise gene module based on GO_CC (A) and GO_MF (B); Purple module gene based on GO_CC (C) and GO_MF (D). Supplementary Table 1 Information of 140 patients with oral squamous cell carcinoma Supplementary Table 2 The correlation of OSCC clinical‐pathological variables Supplementary Table 3 13 color genes Number of genes contained in the color module [file CAM4-12-19260-s001.docx]
